# Climate smart Dry Chain Technology for safe storage of quinoa seeds

**DOI:** 10.1038/s41598-020-69190-w

**Published:** 2020-07-28

**Authors:** Muhammad Amir Bakhtavar, Irfan Afzal

**Affiliations:** 1 Department of Agronomy, Muhammad Nawaz Shareef University of Agriculture, Multan, 66000 Pakistan; 2 Seed Physiology Lab, Department of Agronomy, University of Agriculture, Faisalabad, 38040 Pakistan

**Keywords:** Plant sciences, Plant physiology

## Abstract

Quinoa (*Chenopodium quinoa*) is a climate resilient crop having superior nutritional profile compared to other cereal grains and may help to ensure future food security. Commercial cultivation of quinoa is dependent upon availability of quality seed. Adoption of Dry Chain Technology: drying before storage and maintaining seed dryness through hermetic packaging, may prevent quinoa seed deterioration. Quinoa seeds were dried to 8, 10, 12 and 14% initial seed moisture content (SMC) and stored in conventional (Paper, polypropylene, cloth and jute) and hermetic Super Bags for 6, 12 and 18 months. Seed stored in Super Bag at 8% initial SMC maintained low seed moisture and higher germination. Total soluble sugars and α-amylase activity were higher while EC, reducing sugars and MDA contents were low for the seeds stored in hermetic bag at 8% initial SMC. Seed stored in traditional packaging materials irrespective of initial seed moisture contents, gained moisture due to ambient high relative humidity which resulted in seed deterioration as indicated by increased reducing sugars, MDA contents and seed leachates conductivity and reduced vigor, viability, soluble sugars. The Dry Chain Technology preserves seed quality by maintaining low seed moisture and reducing deteriorative physiological and biochemical changes in the quinoa seed.

## Introduction

Quinoa is an important crop selected by WHO which may ensure future food and nutritional security. UNO celebrated year 2013 as “International Year of Quinoa” with a chanted slogan “A future sown thousands of years ago”^1^. Its protein quantity and quality is better than traditional cereals due to presence of all essential amino acids. Furthermore, higher quantities of minerals (Ca, Fe, Zn) along with “healthy supportive fatty acid profile” (omega 3 fatty acid) makes it an excellent super food^2^.

Quinoa was restricted to its native countries up to 1980 but now quinoa has expanded all over the world with its cultivation in more than 100 countries^3,4^. Successful commercial cultivation of any crop depends upon availability of quality seed. Quinoa seed has higher oil contents compared to cereals such as corn^5^, which suggests the need of special care during production and storage to make available quality seeds at the time of sowing. Quinoa seed contains both perisperm and reduced endosperm, embryo consisting of hypocotyl radicle axis and two cotyledons, seed coat having pericarp on outer side^6^.

Quinoa seed absorbs and desorbs water very quickly due to porosity in integuments and loses viability very quickly^7^. In quinoa seed, moisture gain can be used as indicator to predict seed longevity^8^. Seed stored in conventional packaging materials gains moisture from the environment^9^ when external relative humidity is higher which is normally prevailing in the farmer’s seed stores during monsoon season in developing countries^10^. Quinoa seed imbibe water quickly due to small hole in the middle of the seed, far from the micropyle and there is no interference with absorption of water from the surroundings^11^. At high seed moisture contents increase metabolism of soluble sugars and higher rate of Maillard reaction has been reported in seed of *Vigna radiata*^12^.

Quinoa originated from the Andean Mountains and its adaptation in tropics at low altitude having high temperature is challenging for the researchers. Thus, its seed quality is very tricky after harvest to next growing season especially in high relative humidity and temperature prevailing environment^13^. Quick decline in viability of quinoa seed after harvest to next growing season emphasized to investigate the physiological and biochemical changes associated with seed longevity under ambient storage conditions. Moreover, this crop is gaining massive importance yet there is no on farm seed storage technology and packaging material available to the farmers in the developing countries. Reducing seed moisture contents through drying has been an important strategy to prevent the seed deterioration. The Dry Chain Technology, proposed in this study identified high relative humidity as major factor that could accelerate the deterioration of stored commodities^14^. Unlike cold chain which requires continuous refrigeration, the dry seeds can be stored at ambient temperature using Dry Chain thus reducing the energy requirement for short term safe storage. Seed drying can be carried out either thorough sun drying or using desiccants such as drying beads. Farmers can easily adopt this technology as it is simple, economical and require no energy for refrigeration. In this study, packaging materials have been optimized for short term storage of quinoa seed in relation to the moisture variation and associated physiological changes. In Dry Chain Technology, product dryness is maintained during storage by use of hermetic packaging such as Super Bag. Super Bags are made up of triple layer of poly have very low oxygen (< 4 ccm^−2^ day^−1^) and water vapor transmission rate (< 5 gm^−2^ day^−1^) due to gas coated barrier layers^15^. Prospects of Dry Chain Technology have been explored in this study to maintain seed quality and to prevent deterioration during different storage durations at ambient temperatures by using different packaging materials.

## Results

### Seed moisture contents

**Figure 1:**
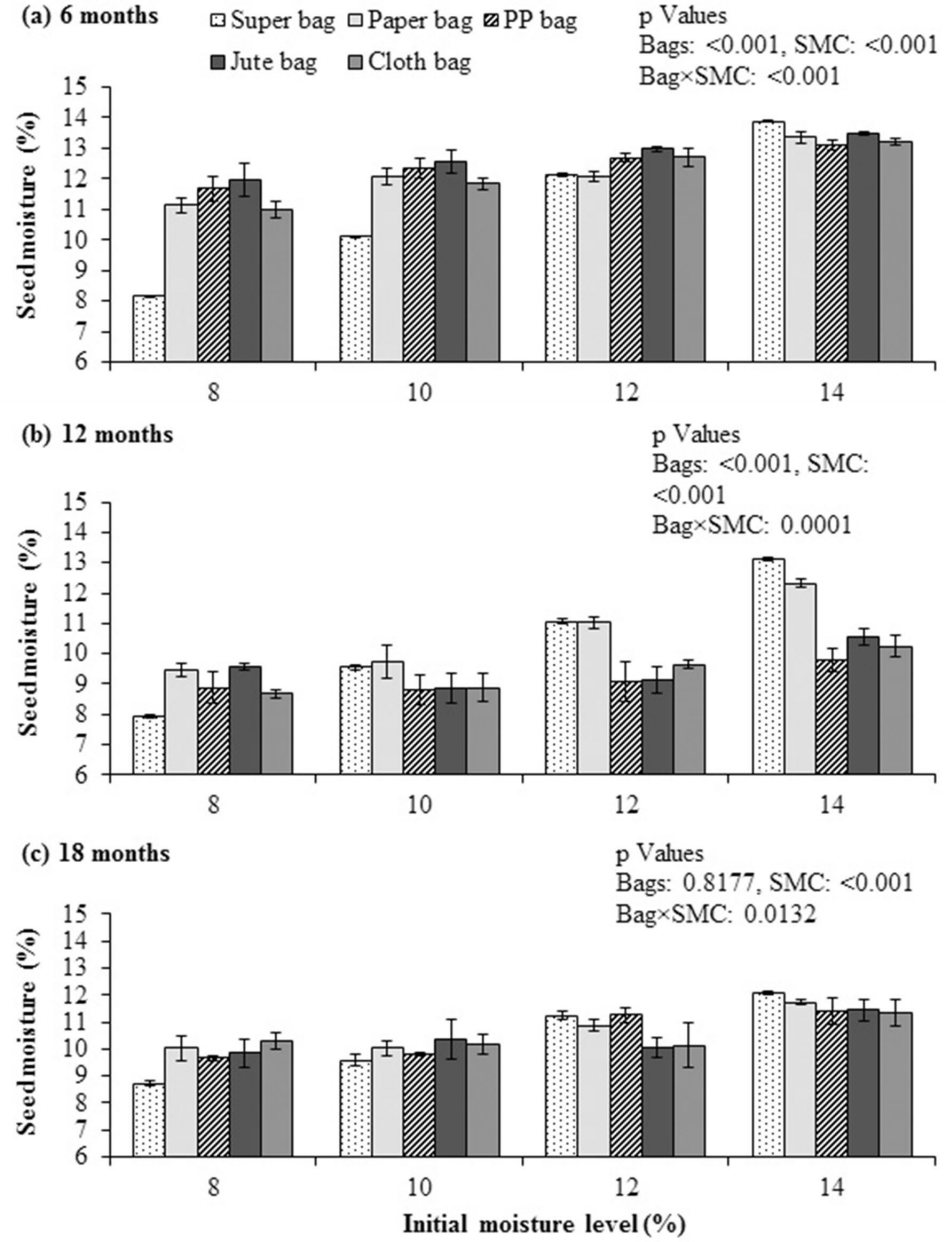
Final moisture contents of quinoa seed after 6 (**a**), 12 (**b**) and 18 (**c**) months of storage at various seed moisture levels in different packaging materials.

Quinoa seed moisture contents after six months of storage were significantly (*P* ≤ 0.05) lower in Super Bag having seed at 8% initial SMC. Moisture contents stayed near to the initial in Super Bag (Fig. 1a). Maximum moisture contents were measured for the seeds stored in Super Bag and all conventional packaging materials at 14% initial SMC and in PP bag and jute bag at 12% initial SMC (Fig. 1a). In conventional packaging materials especially for the seeds which were initially dried to 8 and 10% (28.2 and 40.4% eRH respectively), SMCs increased to 12%. The eRH of seed in conventional packaging materials increased up to 55% from initial 28.2% due to high ambient RH (Fig. 8).

After 12 months, maximum seed moisture contents were recorded for the seeds stored in Super Bag at 14% SMC and in paper bag at 14% initial SMC. Quinoa seed stored in Super Bag at 8% initial SMC have lowest (8.17%) seed moisture contents (Fig. 1b). Overall seed moisture dropped in all bags having seed at 12 and 14% initial SMC due to low RH in storage environment. After 18 month of storage, quinoa seed in Super Bag at 8% initial SMC had lowest seed moisture contents (8.73%) while seed stored in paper, PP, jute and Super Bags at 14% initial SMC had maximum moisture contents. Super Bag retained nearly initial seed moisture for all moisture levels. Seed stored in PP and paper bags at 12 and 14% initial SMC gained moisture from its surroundings (Fig. 1c).

### Germination

**Figure 2:**
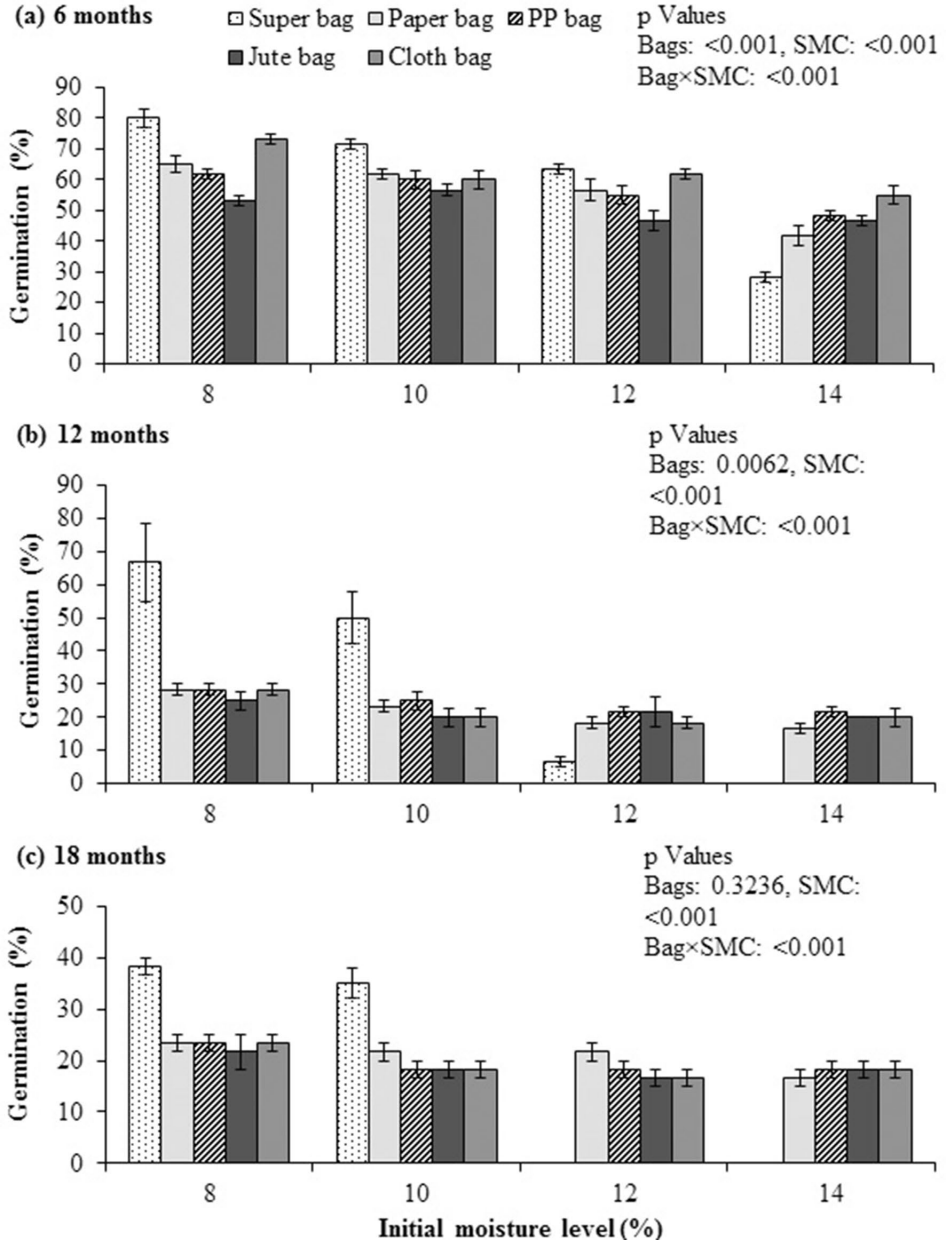
Germination of quinoa seed after 6 (**a**), 12 (**b**) and 18 (**c**) months of storage at various seed moisture levels in different packaging materials. Initial germination 80.5%.

Quinoa seed storage in Super Bag at 8 and 10% initial SMC and in paper bag at 10% SMC resulted into highest germination (80%) after 6 months. Lowest germination (28%) was exhibited by quinoa seeds stored in Super Bag at 14% initial SMC (Fig. 2a). Similarly, germination also dropped significantly in conventional bags. After 12 months of storage in Super Bag at 14% initial SMC, quinoa seed completely lost its germination (Fig. 2b). More than 50% reduction in germination was observed in all conventional bags irrespective of the initial moisture contents. Maximum germination was recorded of seeds that were stored in Super Bag at 8 and 10% initial SMC. Similarly, after 18 months, there was no germination observed for the seeds stored in the Super Bag at 12 and 14% initial SMC (Fig. 2c). Highest germination was recorded for seeds stored in Super Bag at 8 and 10% initial SMC.

### Seed vigor

**Figure 3:**
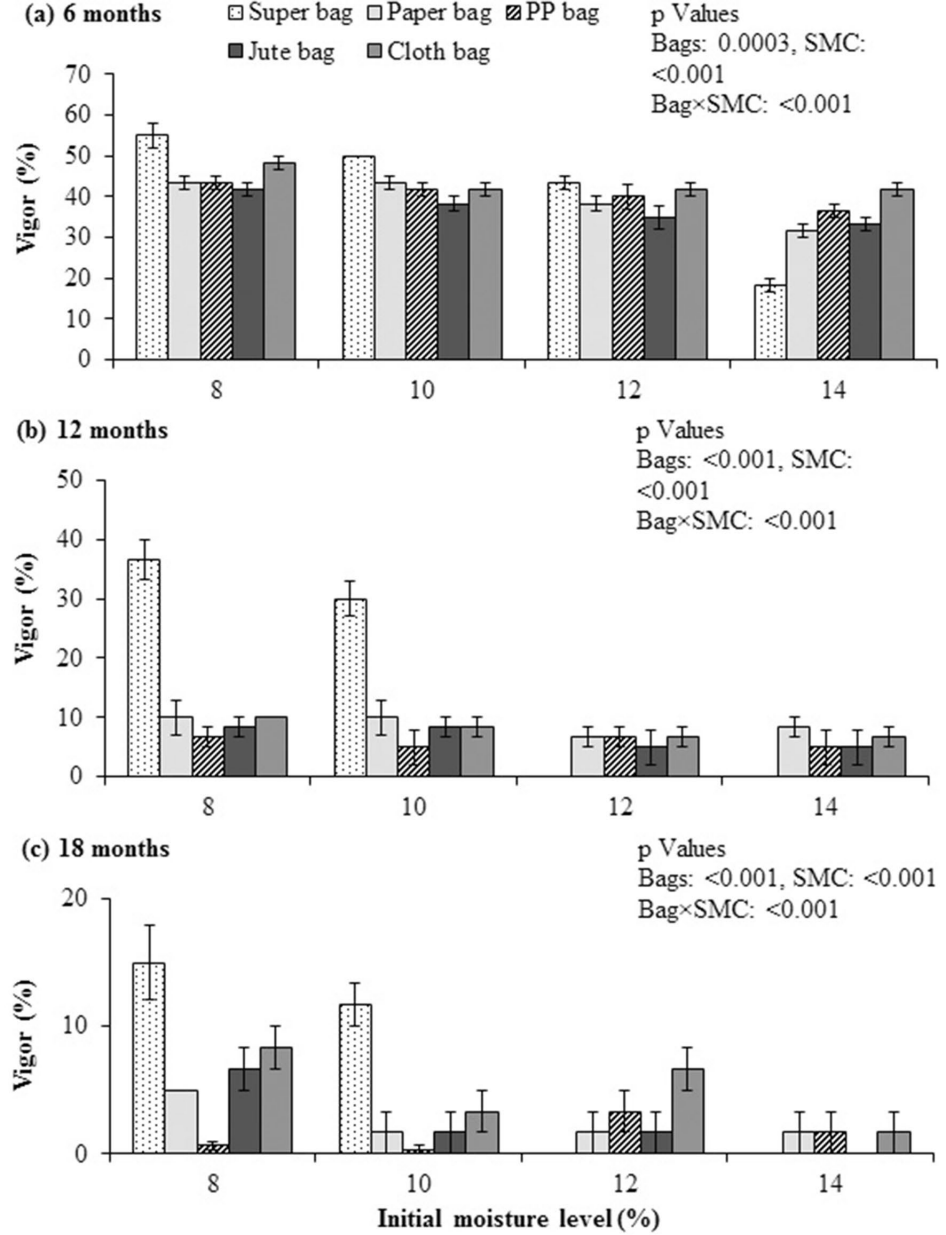
Vigor of quinoa seed following accelerated aging test after 6 (**a**), 12 (**b**) and 18 (**c**) months of storage at various seed moisture levels in different packaging.

Quinoa seed subjected to accelerated aging, after sampling at 6, 12 and 18 month of storage showed a significant difference in vigor of seeds stored in various packaging materials at all four levels of initial SMC (Fig. 3a). After 6 months, quinoa seeds stored in Super Bag at 14% initial SMC had minimum percent vigor. Seeds stored in Super Bag at 8 and 10% initial SMC and in cloth bag at 8% initial SMC had maximum vigor (Fig. 3a). After 12 and 18 months, vigor of quinoa seed was higher for the seeds stored in Super Bag at 8 and 10% initial SMC while seed vigor was completely lost by the seeds stored in Super Bag at 14% initial SMC (Fig. 3b,c).

### Electrical conductivity of seed’s leachates

**Figure 4:**
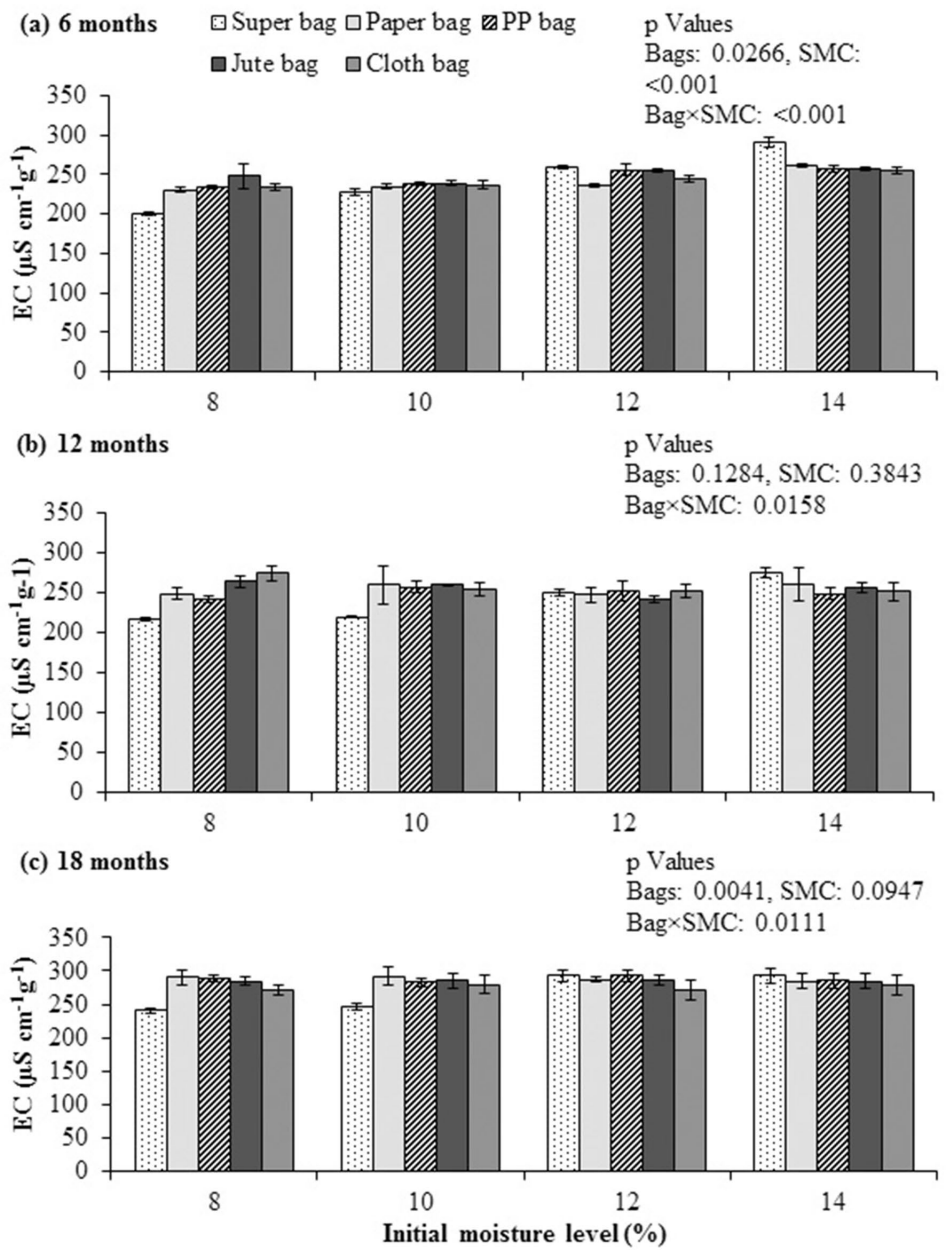
Electrical conductivity (EC) of quinoa seed after 6 (**a**), 12 (**b**) and 18 (**c**) months of storage at various seed moisture levels in different packaging materials.

Electrical conductivity of quinoa seed leachates was substantially lower when stored in Super Bag for 6 months at 8% initial SMC. Electrical conductivity of seed leachates was higher for seed store in Super Bag at 14% initial SMC (Fig. 4a). Similarly, seeds stored in conventional bags also have higher EC indicating rapid deterioration due to high moisture (61% eRH and 13.5% SMC). After 12 months, seed stored in Super Bag at 8 and 10% initial SMC had markedly lower values of seed leachate’s EC. Seed leachate’s EC was more for the seeds stored in porous packaging materials (paper, PP, jute and cloth) at 8, 10, 12 and 14% SMC and in Super Bag at 12% initial SMC (Fig. 4b,c).

### Biochemical attributes

**Figure 5:**
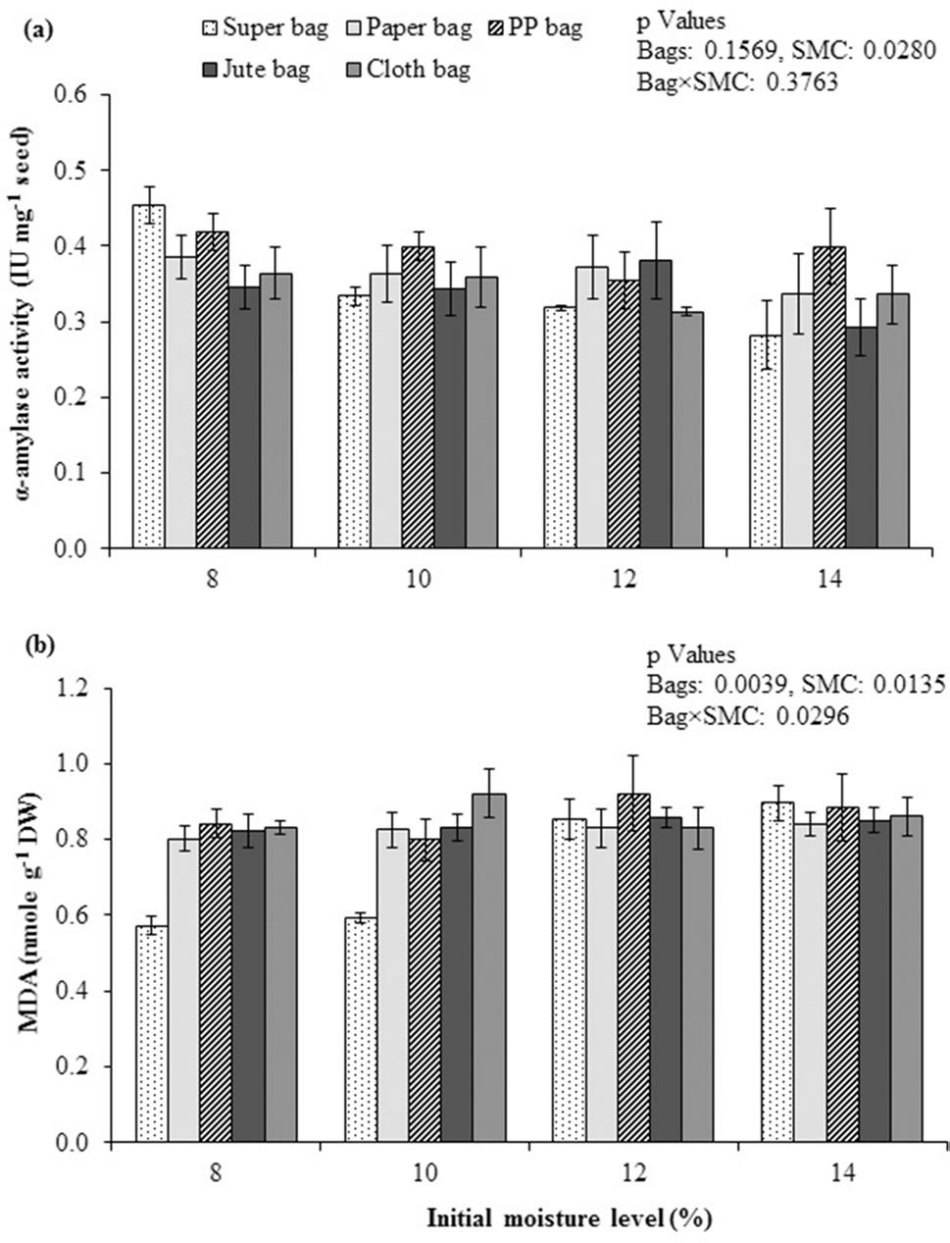
α-amylase activity (**a**) and malondialdehyde (MDA) contents (**b**) of quinoa seed stored at various seed moisture levels in different packaging materials.

The α-amylase activity was not significantly different for all types of bags at 8, 10, 12 and 14% initial SMC. Quinoa seeds stored in hermetic Super Bag showed maximum α-amylase activity (Fig. 5a) showing effectiveness of maintaining low seed moisture in hermetic bags. Malondialdehyde (MDA) contents were minimum in seeds that were stored in Super Bag at 8 and 10% initial SMC indicating protection against lipid peroxidation in dry seeds. Seeds stored in rest all types of packaging materials at all levels of initial SMC had higher MDA contents (Fig. 5b). Overall MDA contents were positively related to seed moisture contents i.e. higher the seed moisture contents higher were the MDA contents and lipid peroxidation.

**Figure 6:**
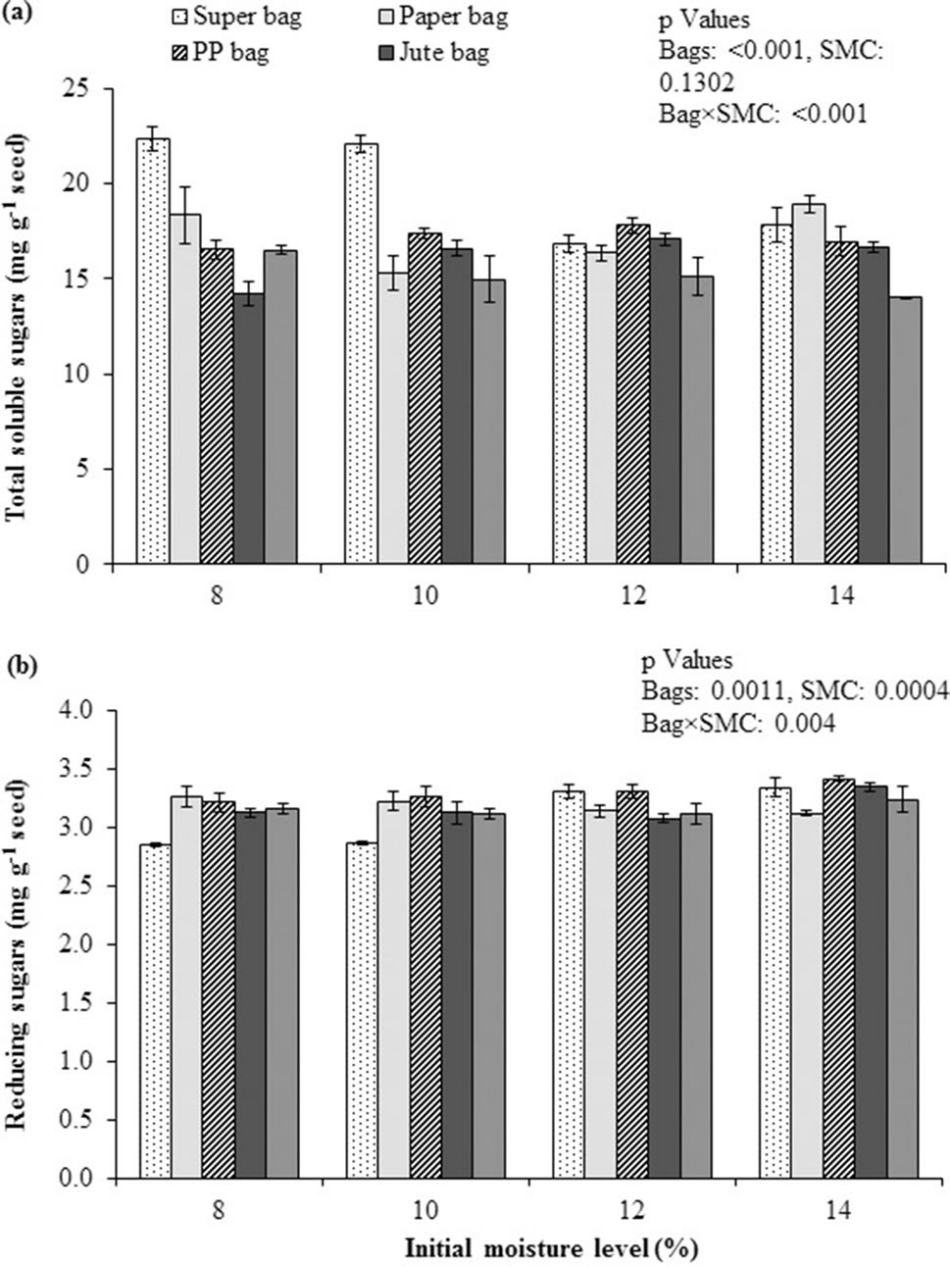
Total soluble sugars (**a**) and reducing sugars (**b**) of quinoa seed stored at various seed moisture levels in different packaging materials.

Maximum quantity of total soluble sugars (TSS) were measured from seeds stored in Super Bag at 8 and 10% initial SMC. Lower TSS were quantified from seeds stored in cloth bag at 14% initial SMC (Fig. 6a). Lowest reducing sugars were present in seeds that were stored in Super Bag at 8 and 10% initial SMC while quinoa seeds stored in rest of the packaging materials at all levels of initial SMC had highest reducing sugars (Fig. 6b).

## Discussion

**Figure 7:**
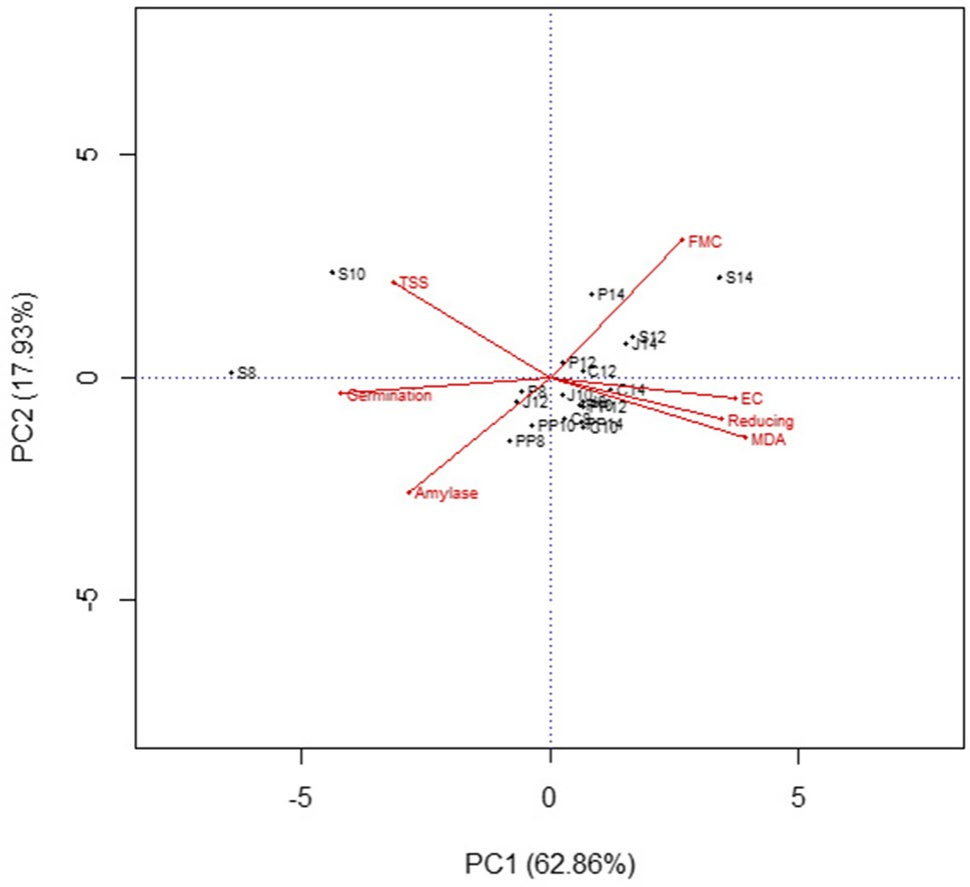
Biplot of germination, EC and various biochemical attributes of quinoa seeds stored in different packaging materials at varying levels of seed moisture contents. S, Super Bag; P, Paper bag; PP, Polypropylene bag; J, Jute bag, C, Cloth Bag. Initial moisture levels: 8, 10, 12 and 14%.

Seed moisture content contributes significant role in all biological and metabolic activities occurring within the living seeds, which modulates seed behavior either towards longevity or deterioration during storage. Biplot analysis indicated that seed germination, total soluble sugars and α-amylase activity were negatively correlated with final seed moisture contents and positively correlated with EC, MDA and reducing sugars (Fig. 7). Overall germination, vigor and seed quality were higher for seeds stored in Super bag at 8 and 10% initial SMC. Hermetic nature of Super Bag hindered both moisture entry and exit into the seeds as it has very low (≤ 5 gm^−2^ day^−1^) water vapor transmission rate^15^. Moreover, lack of fresh oxygen supply due to very low oxygen transmission rate in Super Bag decreased deteriorative changes in seeds. Seed moisture contents in porous packaging materials varied with the ambient RH due to hygroscopic nature of seed^16^ and seed quality was damaged under high seed moisture contents.

**Figure 8:**
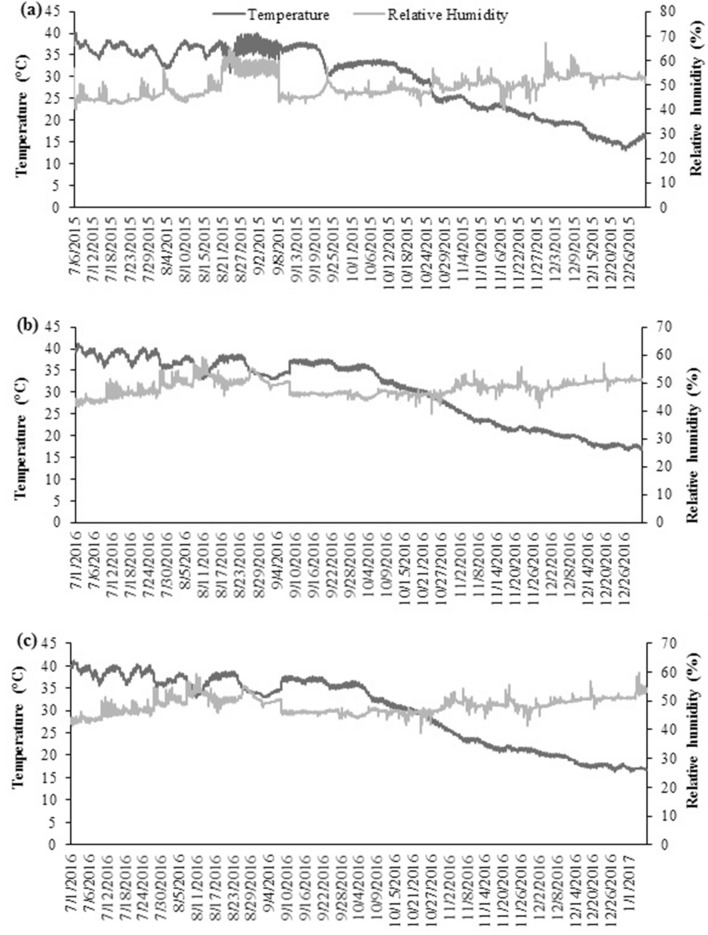
Data of daily temperature and relative humidity of seed store room.

With the passage of time germination decreased (almost 60% reduction in conventional bags and at 14%) as is evident from the germination data after 6, 12 and 18 months of sampling but that decline was slow in Super Bag at 8% initial SMC. High germination of seed stored in Super Bag at 8% initial SMC showed a negative relation with seed moisture contents and deteriorative process (Fig. 7). Previous well-known fact is that each 1% reduction in seed moisture contents doubles the storage life of the seeds^17^. Seeds stored in Super Bag at 14% initial SMC quickly lost their germination as Super Bag continuously maintained high moisture contents near to initial seed moisture contents i.e. 14% due to gas coated barrier layers. Seed germination declined in all conventional packaging materials indicating almost similar moisture gain and aging pattern in these bags. Decline in germination of the seeds stored in porous packaging materials can be attributed to high seed moisture contents that lead to the production of reactive oxygen species (ROS) causing seed deterioration^18^. Seeds in conventional bags gained moisture from their surroundings as ambient RH approached to 70% during some part of the year (Fig. 8). Dry seeds are unable to respire as there is limited biological activity at such low water activity. Seeds at high moisture contents continue to respire and lost viability when there is free supply of oxygen but under limited oxygen supply seeds continue anaerobic respiration and ethanol is produced causing seed deterioration^19^. So seed deterioration and ethanol production are most likely the reasons for seed deterioration and viability losses in Super Bags at 12 and 14% seed moisture contents.

Rate of seed vigor and viability losses are the functions of seed moisture contents and temperature of storage environment^20,21^. Seed leachates electrical conductivity was low after first 6 months and increased with the passage of time, which confirms that deterioration is a continuous process, and with the passage of time seed loses its vigor and viability due to deterioration. Higher the value of the seed leachate’s EC, higher will be the seed deterioration and lower will be the seed viability and vigor. With passage of time, seed leachate’s EC generally increased during storage indicating the process of seed aging and loss of membrane integrity^22^.

Lipid peroxidation is a key process involved in seed aging and malondialdehyde (MDA) contents are the end product of this deteriorative process. Higher MDA contents were present in quinoa seed stored in conventional porous bags as compared to Super Bag (Fig. 5b) as seed in conventional bags gained moisture from its surroundings. High seed moisture contents resulted into higher activities of ROS in the seeds that resulted into production of MDA through lipid peroxidation^18^. However, at low moisture contents role of secondary ROS (Hydroxyl radical and hydroperoxides) cannot be excluded which are involved in seed’s cell deterioration through autoxidation processes. Use of available oxygen for aerobic respiration by dry seeds is very low and is almost absent in some cases. The oxygen absorbed by the seeds in such dry conditions is most likely is related to formation of ROS that are involved in oxidation of biomolecules such as DNA, lipids and phospholipids^19^. Hermetically sealed Super Bags have very low oxygen permeation rate that restricted the free access of oxygen to the stored seeds hence reduced the seed deterioration due to ROS attack. Seed storage in anoxia have been proposed as an effective strategy to protect lettuce germplasm from deteriorative activities and viability losses^19^. Low oxygen and high carbon dioxide concentrations within the Hermetic PICS bags have proven to provide protection against storage insect pests and viability losses in sorghum^23^. Use of hermetic bags under anoxia may offer another opportunity to preserve seed quality. At higher moisture contents use of Super Bag with only twist and tie approach badly affect seed quality due to anaerobic respiration and ethanol production^19^. However, research is needed to study the practical applications of creating anoxia in hermetic bags for commercial scale seed storage.

Deteriorative biochemical changes in quinoa seeds were linked with high seed moisture contents in conventional packaging materials. Higher α-amylase activity indicates vigorous seeds with less deterioration. Reduced α-amylase activities^24^ and total soluble sugars have been observed in aged seeds^25^. Total soluble sugars were maximum in the seeds that were stored in the Super Bag at 8 and 10% initial SMC, indicating slow speed of metabolic activities in the seeds having low moisture contents. Seed deterioration in porous packaging materials resulted into decreased total soluble sugars as was observed in wheat seeds in previous studies^26^. The reasons for reduced total soluble sugars are higher metabolism rate in respiring seeds at high seed moisture contents and Maillard reaction^12^. Studies have revealed the consumption of starch reserves in endosperm and high malondialdehyde contents in primed rice seed at high relative humidity which is the driving force for increased seed moisture contents^27^. Reducing sugars were formed as result of sugar hydrolysis under high seed moisture contents^12^. Moreover, ROS are produced due to aging that react with sugars and convert them into reducing sugars which interfere with the normal functioning of membranes.

## Conclusion

Dry quinoa seeds can be stored for longer period under ambient conditions in hermetic packaging like Super Bag. Seed deterioration occurs more rapidly due to slight increase in seed moisture contents that triggers harmful physiological and biochemical changes in the seed. Seed drying to low moisture is a prerequisite for storage in hermetic bags, as is evident from deterioration of seed stored in hermetic bags at higher moisture contents which is most likely due to anaerobic respiration and ethanol production. Seed stored in traditional packaging materials gained moisture due to ambient high RH, which resulted in deterioration.

## Methods

### Experimental and treatment details

**Figure 9:**
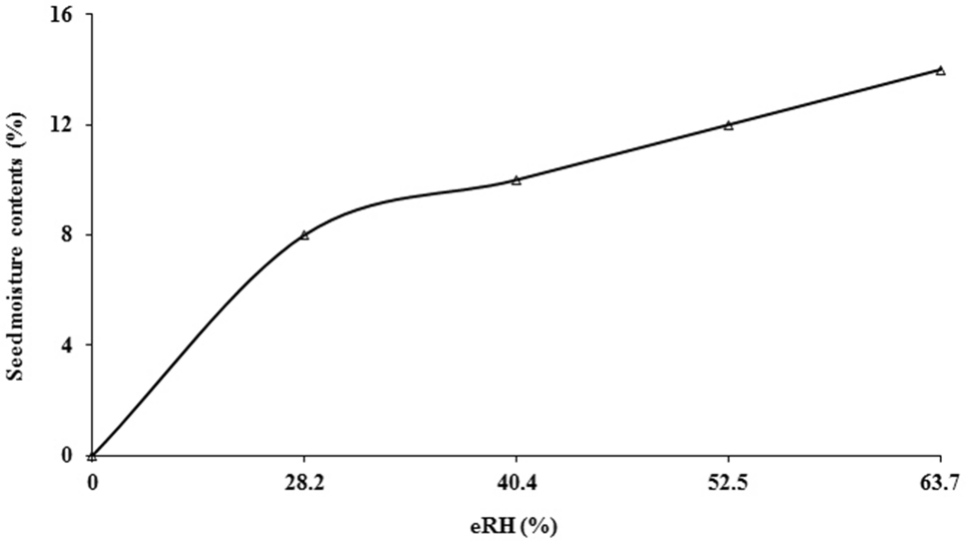
Moisture isotherm of quinoa seed.

Experiment was performed in Seed Physiology Lab, University of Agriculture Faisalabad, during July, 2015 to January 2017. Seeds of well adapted quinoa genotype of Pakistan; UAF Q-7^28^ was obtained from Alternate Crops Lab, Department of Agronomy, University of Agriculture Faisalabad, Pakistan. Initial seed germination and moisture contents were 80.5% and 10.2% respectively. Conventional bags used in this study including woven polypropylene (PP bag), paper, cloth and jute bags were purchased from local grain market. Super Bags of 10 kg capacity were obtained from GrainPro Inc. Philippines. After equilibrating seed to four different moisture contents i.e. 8, 10, 12 and 14% moisture isotherms were drawn by using equilibrium relative humidity (eRH) values at 28 °C. Quinoa seed, having 6.5% oil contents has 28.2, 40.4, 52.5 and 63.7% eRH at 8, 10, 12 and 14% seed moisture contents (Fig. 9). After that three replicates of 5 kg seed were packed in Super Bag and conventional packaging materials making total 180 experimental units (three replicates of each bag at all moisture levels and sampling intervals) and then stored up to 18 months. Relative humidity and temperature of the storehouse were recorded during the whole period of study with the help of Data Logger (Fig. 8). Seed sampling was done after every six months from each of 60 bags. Seed samples were subjected to germination, vigor and electrical conductivity test. Biochemical attributes were determined at last sampling after 18 months of storage.

### Seed drying and equilibrating seed moisture contents

Zeolite drying beads were mixed with seed in airtight plastic boxes to dry seed up to 8 and 10% moisture contents. Seed drying beads (Rhino Research, Thailand) are made up of aluminium silicate clay having very small, uniform pores where water molecule can be adsorbed and are able to dry seeds very quickly^29,30^. Total 579 g drying beads were added in 5 kg seed to lower down moisture contents from 10.2% to 8% at drying temperature of 30ºC. Similarly, to dry down seed from 10.2% up to 10% seed moisture contents, 50 g drying beads were mixed with 5 kg seeds^14^. Oven dry method for determination of seed moisture contents was used to calculate the amount of water required to raise the seed moisture content up to 12 and 14% using following equation:

$$ {\text{Amount}}\,{\text{of}}\,{\text{water}}\,{\text{required}}\,\left( {{\text{ml}}} \right) = \left[ {\left( {\frac{{100 - {\text{Initial}}\,{\text{SMC}}}}{{100 - {\text{Final }}\,{\text{MC}}}}{ } \times {\text{Seed}}\,{\text{weight}}} \right) - {\text{Seed }}\,{\text{weight}}} \right] $$

In order to increase seed moisture from 10.2% (initial moisture contents) to 12%, 12 ml water was added drop wise with continuous stirring for thorough mixing. Similarly, 22 ml water was added to increase the moisture from 10.2 to 14% SMC. After drying when seed reached to desired moisture levels, seed was packed in selected packaging materials. After filling the seed in Super Bag, the upper part of bag was twisted and folded back and then closed with the help of cable tie.

### Seed moisture and germination test

Seed moisture contents were determined using oven dry method by drying weighed amount of seeds at 103 °C for 17 h^31^ and dry weight of each replicate was recorded to calculate the seed moisture contents using following equation:

$$ {\text{Seed}}\,{\text{moisture}}\,{\text{contents}}\,\left( {\text{\% }} \right) = \frac{{{\text{Fresh}}\,{\text{weight}} - {\text{Dry}}\,{\text{weight}}}}{{{\text{Fresh}}\,{\text{weight}}}} \times 100 $$

Germination test was conducted by placing quinoa seeds in sterilized and well moist filter paper following ISTA standards^31^. Seed sample was taken from each replicate bag and from that sample total four hundred seeds were tested in four replicates of 100 seeds. Temperature of the germinator was maintained at 20 °C.

### Determination of seed vigor

Seeds were artificially aged in a climatic chamber (F.lli Della Marca S.r.l. Rome) by placing in seed vigor trays at 45 °C for 72 h. Relative humidity of the chamber was maintained at 95% during this incubation period^32^. Following, accelerated aging, standard seed germination test was conducted as mentioned earlier.

### Measurement of electrical conductivity of seed leachates

From each replicate 50 seeds were counted, and their weight was recorded. Seeds were placed in glass beaker containing 250 ml distilled water of known EC. The glass beakers were covered with aluminum foil and then placed at 20 ± 2 °C for 24 h^31^. After 24 h of soaking, conductivity readings (EC) were recorded with an electrical conductivity meter (HI 99,300). EC of per gram seed’s leachates was calculated using equation following equation:

$$ {\text{Conductivity}} = { }\frac{{{\text{Conductivity}}\,{\text{reading}} - {\text{Background}}\,{\text{reading}}}}{{{\text{Weight }}\,{\text{of}}\,{\text{replicate}}\,\left( {\text{g}} \right){ }}} \times 100 $$

### Seed biochemical attributes

Total 50 g seed was ground to conduct biochemical assays. Activity of α-amylase was measured in 0.5 mg ground seed sample extracted in 5 mL sodium phosphate buffer^33^. For measurement of reducing sugars, 0.5 mg seed was extracted in 5 mL sodium phosphate buffer (pH 6.9) by DNS method^34^. Amount of reducing sugars in samples were calculated from the standard curve prepared from standard glucose solution.

Anthrone method was used to measure total soluble sugars from the 0.5 g seed sample ground in 0.5 mL methanol^34^. Malondialdehyde contents were measure in 0.5 g seed uniformly ground in 10% trichloroacetic acid (TCA). Supernatant was reacted with 0.6% thiobarbituric acid (TBA) prepared in 20% TCA^35^. After incubation, cooling and centrifugation, absorbance of supernatant was taken at 600, 532 and 450 nm to calculate MDA contents using following equation;

$$ {\text{MDA }}\left( {{\text{nmol}}/{\text{g FW or DW}}} \right) = 6.45{ }\left( {{\text{A}}_{532} - {\text{A}}_{600} } \right) - 0.56 \times {\text{A}}_{532} $$

### Statistical design and data analysis

Experiment was laid out in Completely Randomized Design (CRD) with factorial arrangement keeping three replications. Different levels of seed moisture contents and packaging materials were taken as factors. Data were analyzed using statistical software Statistix 8.1. Means are presented in bar graphs along with standard error and p values. R statistical computing software was used to make biplot showing relationship among different parameters of seed quality.
